# Human Growth and Growth Hormone: From Antiquity to the Recominant Age to the Future

**DOI:** 10.3389/fendo.2021.709936

**Published:** 2021-07-05

**Authors:** Evan Graber, Edward O. Reiter, Alan D. Rogol

**Affiliations:** ^1^ DO Division of Pediatric Endocrinology, Nemours/Alfred I. Dupont Hospital for Children, Wilmington, DE, United States; ^2^ Baystate Children’s Hospital, UMassMedical School-Baystate, Springfield, MA, United States; ^3^ Pediatrics/Endocrinology, University of Virginia, Charlottesville, VA, United States

**Keywords:** growth, growth hormone, species specificity, recombinant DNA technology, FDA indications, long-acting growth hormone

## Abstract

Since antiquity Man has been fascinated by the variations in human (and animal) growth. Stories and art abound about giants and little people. Modern genetics have solved some of etiologies at both extremes of growth. Serious study began with the pathophysiology of acromegaly followed by early attempts at treatment culminating in modern endoscopic surgery and multiple pharmacologic agents. Virtually at the same time experiments with the removal of the pituitary from laboratory animals noted the slowing or stopping of linear growth and then over a few decades the extraction and purification of a protein within the anterior pituitary that restored, partially or in full, the animal’s growth. Human growth hormone was purified decades after those from large animals and it was noted that it was species specific, that is, only primate growth hormone was metabolically active in primates. That was quite unlike the beef and pork insulins which revolutionized the care of children with diabetes mellitus. A number of studies included mild enzymatic digestion of beef growth hormone to determine if those “cores” had biologic activity in primates and man. Tantalizing data showed minimal but variable metabolic efficacy leading to the “active core” hypothesis, for these smaller peptides would be amenable to peptide synthesis in the time before recombinant DNA. Recombinant DNA changed the landscape remarkably promising nearly unlimited quantities of metabolically active hormone. Eight indications for therapeutic use have been approved by the Food and Drug Administration and a large number of clinical trials have been undertaken in multiple other conditions for which short stature in childhood is a sign. The future predicts other clinical indications for growth hormone therapy (and perhaps other components of the GH?IGF-1 axis), longer-acting analogues and perhaps a more physiologic method of administration as virtually all methods at present are far from physiologic.

## Introduction

Fascination with extremes in the size of man has a long history, even stretching to Antiquity. Giants such as the biblical Goliath confronting David, the Colossus in Goya’s 1808-1812 masterpiece, and the Irish Giants are examples of great height growth, while the diminutive man (likely a person with achondroplasia) in the marvelous 17^th^ century painting of Velazquez, *Las Meninas* and Tom Thumb, a young man with presumed growth hormone deficiency in P.T. Barnum’s shows in the mid-19^th^ century are among several examples of extreme short stature.

Furthermore, more than a century has gone by in which scientists have attempted to understand the metabolic pathways that bring about the wide variations in human growth. One has come from a time of thoughtful animal experimentation to highly sophisticated molecular tools to begin to understand growth. We describe some of the scientific work that has enabled us to begin to address the very basics of the hormonal regulation of growth.

## Overgrowth

### Antiquity

The Old Testament (New International Version) notes the story of David and Goliath in I Samuel 17 (**20-58**):”As the Philistine moved closer to attack him, David ran quickly toward the battle line to meet him. Reaching into his bag and taking out a stone, he slung it and struck the Philistine on the forehead. The stone sank into his forehead, and he fell face down on the ground”. Goliath had gigantism likely secondary to a pituitary tumor that had grown out of the sella turcica to press upon the optic chiasm and cause bitemporal hemianopia with Goliath having tunnel vision (1). The clinical picture of acromegaly may include significant cardiopulmonary disease and osteoarthritis that may be crippling and could have prevented Goliath, with limited mobility, from avoiding the spherical projectile. Additionally, and certainly in the untreated state, there may be skeletal fragility, with the detrimental impact of excessive growth hormone and IGF-1 upon the craniofacial and cervical vertebral skeleton (2) Height estimates for Goliath have varied considerably from four cubits and a span (6 feet 9 inches, ~2.06 m) to 6 cubits and a span (9 feet 9inches, ~2.97 m). The older manuscripts, namely the Dead Sea Scrolls of Samuel, seem to lean toward the lower height measurement (3). Additionally, Goliath or other giants are described as being from the city of Gath (1 Samuel 17.4). In a different war with the Israelites (1Chronicles20:4-8) they were killed by David or by David’s brother and his servants. These giants were said to be descended from a clan (from Gath) of men of large size (4). This raises the question of there being the presence of genetic regulation of the excessive growth hormone production in these men, as has been described elsewhere (see below; Irish Giants). There is mention of the presence of extra digits on hands or feet raising the question of the diagnosis of Bardet-Biedl syndrome, which is inherited closely to an area on chromosome 11q.13 near the gene associated with the inherited pituitary tumor in the Irish giants (Increased population risk of AIP-related acromegaly) (5). On the other hand, their recognition as being a group of vaunted warriors might speak against a growth hormone etiology with all of the health abnormalities of acromegaly.

### Pre-20^th^ Century

In the early 19^th^ century Goya painted *The Colossus*, a dark portrait of a giant towering over a landscape with multiple people in awe of someone of such size; this is displayed in Museo del Prado in Madrid. The medical diagnosis present in this muscular, powerful-appearing man, could include late onset of excessive growth hormone secretion, perhaps from a pituitary tumor, but in a man with abundant androgen production that persists. Alternatively, the painting represents the wishful depiction of a man who has risen to protect the Spaniards from the invading Napoleonic forces during the Peninsular Wars.

The importance of the pituitary for excessive growth was recognized by the French physician, Pierre Marie, who associated the clinical signs and symptoms of acromegaly with an enlarged sella turcica and pituitary tumors (6). Familial gigantism and acromegaly is a fascinating story with roots in the mid-18^th^ century in Ireland (Irish giants). The Irish Giant was a man who was 7 feet 7 inches (2.31 m) tall, named Charles Byrne who achieved fame because of that height, but who died of tuberculosis and chronic alcoholism. His skull was later examined by Harvey Cushing who made the posthumous diagnosis of a pituitary tumor.

Marta Korbonits and co-workers have nicely summarized the story of the Irish Giant in its historical context and defined the molecular defect in the aryl hydrocarbon-interacting protein gene (*AIP*) that causes excessive growth hormone secretion before pubertal maturation and epiphyseal closure, hence gigantism and later acromegaly (7). They studied the DNA of Charles Byrne, 1761-1783, and from other giants from four Northern Irish families. The AIP mutation was found in 10 individuals with growth hormone-secreting adenomas; 8 of 10 presented with childhood-onset disease, and thus excessive height. This mutation was not found in other patients with growth hormone-secreting adenomas from other areas of Ireland. They estimated that the mutation positive pedigrees shared a common ancestor who lived about 2500 years ago (7).

## Under-Growth

Additionally, there have been many descriptions of subjects with severe growth impairment (8). Adelson relates the story of the sexual ateliotic dwarf (old term describing one not yet achieving perfection; in addition the term “dwarf” is now considered pejorative by some (9) and we shall use “little person”). Better known as General Tom Thumb, Charles Sherwood Stratton was born in Connecticut in 1838. –and lived to age 45 years dying of a stroke. His growth pattern from infancy mirrored that of a growth hormone deficient individual. His growth ceased by the middle of the first year of life, not to start growing again until puberty. His adult height was 101.4cm (about 3.5 feet). His appearance and doll-like facies suggest that he had isolated growth hormone deficiency and would have responded well in the present to exogenous growth hormone. He was a long time financially successful member of the P.T. Barnum Circus and was married to a growth hormone deficient woman, with whom he had one child. He traveled the world meeting royalty and President Lincoln.

The lives of little people are explored in great detail by Betty M. Adelson, *The Lives of Dwarfs* (10) [www.hpb.com/products/the-lives-of-dwarfs]. In a masterpiece, *Las Mininas*, produced by Diego Velazquez in the mid-17^th^ century, two little persons are depicted in a remarkable painting of the court of King Phillip IV of Spain with two ladies in waiting and two little persons, one of whom apparently has achondroplasia.

## Finding the Growth-Promoting Agent(S) in the Anterior Pituitary

The presence of a growth-promoting factor in the hypophysis, however, was not demonstrated until Aschner in 1909 employed a buccal approach to the sella to remove the pituitary in puppies. The experimental animals showed severely retarded growth, and poor survival (11). Contemporaneously, Crowe and co-workers found consistently diminished growth in puppies with only partial surgical extirpation of the pituitary or of its anterior lobe (12). By careful postmortem examination they were able to assess the completeness of hypophysectomy and to rule out complicating cerebral injury, hemorrhage, or infection. The older dogs whose pituitaries were completely removed died within 5 days, but some of the puppies remained alive for as long as 20 days; they likely had secondary adrenal insufficiency. Importantly, removal of the posterior lobe had no effect on growth.

By 1912 Aschner and colleagues had perfected the technique of total hypophysectomy and were able to observe cessation of growth in puppies after total hypophysectomy without injury to the adjacent areas of the brain, removing the uncertainty of whether the diminished growth was due to a factor within the pituitary or because of damage to brain structures surrounding the pituitary (13).

It was not until 1921 that a positive effect of the hypophysis on growth could be demonstrated. Evans and Long reported an increase in body weight of *intact* plateaued female rats over their litter mate controls following the intraperitoneal administration of an *aqueous* saline emulsion of bovine anterior pituitary lobes (14). Since murine bony epiphyses never unite, the rats did not have the features of acromegaly, as in the human. “Acromegaly” was first experimentally produced in dogs by Putnam and co-investigators by injection of a sterile aqueous anterior pituitary extract for a period of 14 months. Dogs that received this extract had an increased body weight, enlargement of the acral parts, and polyphagia (15). At autopsy, general splanchnomegaly and skeletal overgrowth with hyperostoses were noted.

Although prior to 1930 certain effects of removal of the anterior pituitary upon growth in animals were known, the post-operative morbidity and mortality in dogs was high and there were no consistently effective methods for performing pituitary surgery in smaller animals. In a now classic series of experiments in 1930, P. E. Smith conclusively demonstrated the necessity of the hypophysis for *normal* growth using a reproducible ventral parapharyngeal approach to the sella in the rat that mitigated some of the injurious adverse events of the trans-buccal approach. Virtually complete cessation of growth followed ablation of the hypophysis or of its anterior lobe. If the posterior lobe alone were removed, there was no effect on growth. It is odd that the likely presence of diabetes insipidus didn’t seem to alter growth. Daily anterior pituitary lobe homoeotransplants restored normal growth patterns-an increase in weight, body measurements and tail length-in the hypohysectomized animals. When injected intraperitoneally, a saline suspension of the bovine anterior lobes also produced physiologic growth, but had no effect on the atrophied reproductive organs (16).

## Extraction and Isolation/Purification of Growth Hormone

Attempts to purify and concentrate the active principle were rewarded with extracts of increasing biological potency with decreasing amounts of contaminating proteins. Although it was clear that the pituitary in some manner controlled growth, since the growth rate could be altered experimentally by pituitary manipulation, no one had isolated a single chemical substance with the specific function of growth promotion. In fact, there were many who believed that such a process as complex as growth could not be controlled by a single chemical factor. Bates and co-workers (17) expressed the general view that since the anterior lobe hormones act upon or through other endocrine glands whose target organs, thyroid, adrenals and gonads, produce hormones for healthy body maintenance, these target glands probably also participate in *normal* body growth. They argued that if one would replace the several hormonal deficits resulting from hypophysectomy, then the animal ought to return to a normal growth pattern. The experimental design used to prove this point consisted of groups of dwarf mice treated with “growth hormone” preparations (contaminated with prolactin and TSH, prolactin (heated to 37°C at pH7.5 to 8 for one hour to decrease the TSH activity and to denature the heat-labile “growth hormone”), and prolactin-free TSH preparations. Animals treated simultaneously with TSH and prolactin gained more weight than the combined weight gain of the TSH and prolactin-treated animals, demonstrating a synergistic effect of these hormones. Since the total weight gain was comparable to that of the rats receiving “growth hormone”, the authors felt that growth was merely the synergistic effect of the pituitary hormones acting through their target organs. It should be noted that all of these experiments were performed with crude mixtures of hormones. Due to this and the fact that they employed mice with a genetically determined growth deficit, it is difficult to draw meaningful conclusions from this study.

In 1938 Evans and colleagues (18) developed a precipitation procedure for the isolation of growth hormone based on earlier methods which had given low yields and were grossly contaminated by lactogenic, thyrotrophic and gonadotrophic factors. Although the product had increased growth-promoting activity, it was accompanied by an increase in the activity of the contaminants. Several years later Frankel-Conrat and colleagues (19) used extraction in a cysteine-containing medium to prepare a more homogeneous product with greatly decreased contaminating hormones, but activity of the adrenals, thyroid and the preputial glands in the rat was still apparent. At approximately the same time Fevold and collaborators were able to prepare five anterior pituitary fractions rich in the individual hormonal activities, but none was chemically or biologically homogeneous (20).

Thus by the mid-1930’s, the pathophysiology of acromegaly as well as the necessity of a factor from the anterior pituitary for physiologic growth were known. The next task was to purify and then identify that factor. Thus, hand-in-hand with devising a purification scheme was the related task of developing assays to help sort the growth-promoting factors from those that did not affect growth. The first usable assay was that of Evans and Long, noted above (14). Later, several modifications of this principle were tried using rats. Marx and co-workers developed a technique which measured the increase in body weight of either normal female rats at 5-6 months (weight plateaued) or rats hypophysectomized at 28-30 days of age. The logarithm of the daily dose (17 doses in 20 days) *versus* the body weight gain, a classical bioassay, was linear within a dose range of 0.25 to 4 mg for the normal rats and far more sensitive (0.03 to 0.48 mg) for the hypophysectomized animals (21).

Even greater sensitivity was found using the epiphyseal growth plate, for regressive changes were noted in the proximal epiphysis of the tibia in immature rats. The finding that these changes could be *reversed* by pituitary extracts formed the basis of a new and highly sensitive bioassay for growth hormone (22). Immature hypophysectomized female rats were injected intraperitoneally for 4 days with pituitary fractions in saline. The tibias were removed, sectioned in the sagittal plane and stained, before measuring the width of the proximal epiphyseal cartilage plate. The plot of the width *versus* the logarithm of the daily dose produced a straight line within a dose range of 5 to 200 µg of the “crude” material (22). This method was extended by Greenspan and co-workers who determined the conditions for maximal response and evaluated its specificity, sensitivity and accuracy (23).

This new procedure remarkably hastened the purification first of animal [bovine and porcine] growth hormones (18) and then the simian and human hormones (24). Next steps included the attempts to use bovine growth hormones (Bennett, and coworkers over a three week nitrogen balance study (25), or a longer trial over several months (26) or digests of them in the human (27, 28). Although some tantalizing data were obtained in multiple trials with a variety of enzyme digested growth hormones, no preparation unequivocally had reproducible activity in man. It should be remembered that porcine and bovine insulins were mainstays in the treatment of patients with diabetes mellitus at that time, so it is not surprising that such animal sources were first utilized.

## Species Specificity

The species specificity issue was evaluated by Knobil and co-workers who found that simian and later human growth hormones produced striking proliferative changes in the costochondral junctions of hypophsectomized Rhesus monkeys, but animal growth hormones did not; nor did they permit the retention of nitrogen in balance studies (29, 30). Other metabolic actions, including the auto-inhibition of secretion by pretreatment with simian growth hormone were noted in the intact and hypophsectomized Rhesus monkey [summarized in (31)]. The specificity of the human and simian activity resides in a single arginine residue in the simian (and human) growth hormone receptor and its cognate binding protein (32). The investigators concluded that incompatibility of Arg^43^ in the human GH receptor with His^171^ in non-primate GH is the major determinant of noted species specificity.

## Active Core Hypothesis

Growth hormone is a pleotropic hormone with biological activity for carbohydrate, lipid and protein metabolism. However, unlike animal insulins or ACTH there was little if any biological activity in man. The biological data from primate growth hormones, the lack of activity of the animal hormones or mild enzymatic hydrolysis of the animal hormones in primates including man, and the activities of fragments of human or animal growth hormones in *some* animal bioassays led to an active core hypothesis (33). This property of species specificity was speculated to be due to a broad diversity in primary structure of growth hormone of various species, but all had the ability to form biologically functional peptides that only required a partial sequence. Once these were “unmasked”, each could exert its specific biological activity. Thus the concept is that each of the growth hormones contains an “active core” (or cores) of amino acid sequence responsible for its multitude of biological actions (33). These studies far antedated recombinant DNA. It was speculated that if the cores were small enough one could synthesize the smaller peptides more readily than the full animal GH molecule and attain metabolic effects, perhaps including growth in man. Partial proof of that concept was the derivation of a peptide from bovine GH that induced glucose intolerance in fasted *ob/ob* mice (34). The same authors followed with a study of the cognate peptide *synthesized* from the human GH molecule (35).

One of the authors (ADR) (36) prepared and sequenced cyanogen bromide fragments from bovine GH that were tested in multiple bioassays [summarized in (33)]. None had more than weak activity. Larger peptides from the human molecule strengthened the core hypothesis. Li (37) digested a homogeneous preparation of hGH with pepsin under mild conditions to an extent of almost 40 percent. Separation from the undigested material was done under non-reducing conditions. There were no differences in activities between intact hGH and the pepsin-altered hormone (at multiple doses) in both the tibial growth plate assay and the pigeon crop sac assay (for lactogenic activity). Taken together with data from partial chymotrypsin and carboxypeptidase digests, it is apparent that the activities of human pituitary growth hormone do not depend on the integrity of the entire molecule. One may *infer* that the activity resides in only a portion of the molecule. That does not mean that the core is *contiguous* because of the two disulfide bonds. Given that this was at a time when there was no evidence for an *independent* pituitary lactogenic factor, Professor Li concluded that it appeared that hGH possesses, as an intrinsic property, all of the biological effects characteristic of animal lactogenic hormones (37).

## Somatomedin Hypothesis and IGF-1

Following the successful development of an immunoassay by Yalow and Berson to measure the concentration of insulin in plasma (38), a similar assay to measure growth hormone was validated soon thereafter (39, 40) and allowed the confirmation of the clinical diagnosis of growth hormone deficiency or over production.

Subsequently, there was steady progression (41) of development initially of bioassays for Somatomedin C (now known as IGF-I) then radioimmunoassay techniques for growth hormone and IGF-l (16).

The classic studies of Salmon and Daughaday (42) in the 19*50’s* began a successful journey to understand the biology of growth hormone and the complex nature of its physiology. In their studies, these investigators demonstrated the need for production of a growth hormone-dependent factor to permit the growth promoting activity of GH. They showed that radiolabeled sulfate (SO4) could be taken up by rat cartilage, but that this process was diminished in hypophysectomized rats and not improved by placing GH into the *in vitro* system. However, serum from normal rats or from hypophysectomized rats treated with GH normalized the sulfate uptake (thus, the name “sulfation factor”). Over time, this factor(s) was isolated and had its amino acid sequence determined. The material had insulin-like activity, but blocked *in vitro* by an insulin antibody (thus, non-suppressible insulin-like activity, or NSILA). Finally, the growth promoting activity largely (though not completely) regulated by GH was clarified and two peptides were designated as being insulin-like growth factors (IGF-I and II) (43–45). Green and colleagues proposed a modification to the original hypothesis, denoting a “dual effector” theory of growth hormone action (46). The hypothesis indicated individual functions for GH and IGF-1 (somatomedin). The former promotes the differentiation of precursor, for example, cartilage cells and the latter leads to clonal expansion. Thus, the original endocrine hypothesis has become one of paracrine/autocrine action. The substantial complexity of this GH-IGF system continues to be assessed.

## Growth Hormone Treatment in the Human

Human growth hormone was finally purified, noted to be separate from human prolactin, although there are some overlapping activities of these similar molecules and employed therapeutically to treat GH deficient children (24, 47–49). In a remarkable 60 y follow-up one of Raben’s original patients was re-evaluated at age 78 y. He received hGH (Raben preparation) in 1956 at age 17 y at which time he was 129.5 cm, had no sexual development and a bone age of 7 yr. Two and one half years of hGH led to an adult height of 168.9 cm or 15.5 cm/y during those 21/2 y (50). He was subsequently treated with thyroid hormone, cortisone acetate, and testosterone. Spermatogenesis with successful conception was induced with hCG and human menopausal gonadotropins. MRI examination of the brain at 78 y revealed a tiny pituitary with absent infundibulum. Combined pituitary deficiency genetic panel did not reveal any clinically relevant variant and serum levels of GH, FSH, LH, and testosterone were undetectable (50). In addition in a tantalizing short paragraph titled: Treatment of adult hypopituitarism Raben noted that an adult woman with hypopituitarism had been fully treated with the agents available at that time-thyroid, ACTH and estrogen, but was not completely “well”. Upon addition of growth hormone “…she noted increased vigor, ambition and sense of well-being” (51). Raben goes on to conservatively speculate, “Observations will be needed in more cases to indicate whether the favorable effect was more than coincidental” (51). In our opinion this is an “Ah Ha” moment for hGH to be effective in the hypopituitary adult and intentionally led to the trials of growth hormone in the adult for a number of purposes the non-GH deficient child, and doping in sport—brought to fruition with the production of recombinant hGH (rhGH).

The effects of growth hormone on growth and metabolism were shown in a group of 75 hypopituitary children by Wright and colleagues (52). They emphasized some metabolic aspects of the hormone in the short term and tried to correlate them to the change in height velocity in the longer term. This was not a new concept since others had previously noted the effects of hGH on metabolism, positive nitrogen balance, mineral retention and glucose intolerance (49, 53, 54). hGH administered to fasting subjects led to a fall in free fatty acids, glucose, α-amino nitrogen within an hour followed by a rise in FFA (54).

## Role of the National Pituitary Agency

By the mid-1960s it was clear that human growth hormone was effective in GH-deficient children and especially helpful for those very young infants with hypoglycemia. This situation led to competition for human pituitary glands obtained at autopsy. To maximize gland collection, the distribution of hGH for clinical investigation and therapy, the National Institute of Arthritis, Diabetes, and Digestive and Kidney Diseases (NIADDK) of the National Institutes of Health (NIH) and the College of American Pathologists formed the National Pituitary Agency, NPA (since re-named National Hormone and Pituitary Program, NHPP) which became responsible for all pituitary hormone-related therapy and many reagents for clinical and basic science research (55). A similar program was begun in Canada by the Canadian Medical Research Council.

The NPA was organized under the direction of Dr. Robert Blizzard along with others, Drs. Alfred Wilhelmi, Al Alberts, and several from the NIH (NIAMDD) with the purpose to coordinate the collection of human pituitaries, the extraction and distribution of hGH in a logical and sensible manner for both research and treatment. After a few years of private funding, the NIH provided for the NPA beginning in 1963. The methods for extraction and purification changed during the hGH program with “clinical grade” hormone moving from 1 to 3 IU/mg (essentially pure 22 kD monomeric hGH) (56). Until 1985 when the first few cases of Creutzfeldt-Jakob disease were discovered virtually all of the hGH distributed in the US came from this program. All patients under treatment were part of various research studies for at least the first part of their treatment program which was often interrupted since there was not enough hormone for all patients for each year. One learned a lot about catch-up and catch-down growth in those with hypopituitarism because of this problem (52). It is purported that human pituitary growth hormone added 17.7 km (~11 miles) to the heights of growth hormone deficient children in the US during the NPA distribution period (Blizzard, RM, personal communication).

## Summary (Pre-Recombinant Era)

The activity of growth hormone in humans has been known since at least Biblical times and then through the centuries with many giants/those with acromegaly noted in paintings (e.g., Goya’s *Colossus*). It was not until the late 19th century that acromegaly was considered due to a pituitary tumor. A number of animal experiments were performed in the early 20th century that unequivocally noted a growth promoting hormone from the anterior pituitary, with growth hormone finally purified from the human in the late 1940s. Animal growth hormones were not active in the human (unlike insulin) and the first report of an adolescent with growth hormone deficiency treated with human growth hormone was noted in 1958. Growth hormone was in short supply until the recombinant era when clinical research efforts were expanded to many other conditions for which short stature was a sign in children/adolescent and abnormal body composition and diminished quality of life were signs and symptoms in adults with growth hormone deficiency.

## Recombinant DNA Era

The story of the development and use of recombinant human GH (rhGH) is one of good fortune. Prior to 1982, peptide hormone therapy was relegated to the processing of animal or human cadaveric glands to extract, purify, and subsequently administer therapies such as insulin. As noted previously, because of species specificity of GH, only simian or human GH are metabolically effective in man. Pituitary hGH was used experimentally, but sparingly because of low supply, for many conditions that resulted in short stature. It showed much promise until several reports of Creutzfeldt-Jakob disease (CJD) emerged in 1985.

The description of the dramatic story of the occurrence, clinical presentation and ultimate demise of the index case of GH-associated CJD was told in clear and moving prose by the involved pediatric endocrinologist (57) ten years after the diagnosis. Within 6 months of presenting with ataxia, he had died. An autopsy concluded that he had had CJD (57, 58). Soon thereafter, Drs. Blizzard and MacGillivray described two similar cases (59). Many more followed (60) in the US and throughout the world, especially in France (61, 62). Distribution of pituitary-derived-GH (cadaveric) was swiftly halted in the United States and most of Europe in 1985 because of concern about a causal relationship with CJD, a fatal spongiform encephalopathy that had been previously reported to be capable of iatrogenic transmission through human tissue. To date more than 250 young adults who had received human cadaveric pituitary products have been identified with CJD with the sad likelihood that all affected patients will die of the disease. In the US, the onset of CJD is 14 to 33 years after starting cadaveric GH, while the large cohort of French patients had a median incubation of approximately 5 years less.

Vigilant NIDDK surveillance for this dreadful complication continues, although the incubation period would now be as long 40 years and hopefully at its conclusion [https://www.niddk.nih.gov/health-information/endocrine-diseases/national-hormone-pituitary-program/comprehensive-report]. An alternative treatment was clearly needed. This led to the whole saga of biosynthetic GH and the revolution in treatment of children and adults with rhGH.

Almost by design, recombinant DNA technology for GH developed at the same time that pituitary GH was being extracted and supplied by the NPA, as discussed in the previous section. In 1972, the first study demonstrating the use of recombinant DNA technology was published (63). By 1979, messenger RNA procured from pituitary tumors was used to reverse transcribe the hGH gene, which was subsequently inserted into the genome of *E. coli*. Two collaborating groups reported that these transfections resulted in production of GH protein that was biologically active in man (64–66). Once it was shown that GH could be produced safely and in mass quantities, the age of rhGH therapy began. Here, we review the history of the currently FDA-approved uses of rhGH in children and adults and *focus* on those studies that were pivotal to specific indications, some published after the FDA approval, but whose data were used in the decision to approve.

## Growth Hormone Deficiency (1985)

The first product was methionyl growth hormone for at the time it was easier to produce and purify a form of the hormone with an additional methionyl group at the amino terminus of the molecule. Although the data for approval first appeared in the FDA submission file, many were published in the results of a clinical trial in 1986 (67). The results showed that the product was as biopotent as the pituitary formulation to promote linear growth. It had the same metabolic effects (reducing blood urea nitrogen, increasing the serum phosphorous and alkaline phosphatase and raising the concentration of somatomedin C (IGF-1) (67). It had no more diabetogenic activity that pituitary hGH (67). After approval for pediatric GH deficiency studies were undertaken in many other forms of childhood short stature. Subsequent FDA approvals (pediatric) were noted for some (see below). Other conditions which included short stature were subjected to clinical trials, but did not achieve FDA approval. A few of the more prominent are outlined below.

## Chronic Renal Insufficiency (1993)

Although hGH had been used experimentally to treat patients with various conditions that included short stature prior to 1985, the advent of rhGH allowed for larger case series and formalized studies to determine in which conditions children may actually benefit from use. Starting in the 1970’s, several groups started to posit the etiology of growth faltering in children with chronic renal disease, for some of the children are particularly prone to develop severe growth slowing and short stature. Inadequate nutrition may play a role, but there were hints that low somatomedin C (IGF-1) activity could also be an important contributing factor (68, 69). By the end of the decade children were undergoing GH stimulation tests that suggested that GH deficiency was likely not the cause of their slow growth, but rather GH resistance may be the underlying factor (70). Could “flooding the system” with rhGH be the answer to permitting children with CRI to grow more quickly?

Case series demonstrating rhGH administration to children with CRI would continue to be published through the 1980’s, but it would be the 1990’s that would finally bring published clinical trials that suggested that rhGH may be effective in increasing growth in this population. Hoekken-Kolega and colleagues performed a placebo-controlled, cross-over study in 16 children with CRI. Height velocity increased significantly when the patients received rhGH when compared to placebo. Although they were not followed to adulthood, it was concluded that adult height would likely be increased as well, since the bone age was not accelerated by treatment (71). The Kabi Pharmacia study group in Europe (KIGS) made similar conclusions after observational data were obtained from study participants demonstrating significantly increased height velocity after 2 years of rhGH treatment (the rate decreased in the second year, but was still above baseline) (72, 73). By 1993, the data were strong enough that rhGH was approved by the FDA for use in short children with CRI. Additional data have continued to emerge since approval and a consensus statement supporting the use of rhGH in this population has since been published (74).

## Turner Syndrome (1996)

Henry Turner first described 7 women with “infantilism,” webbed neck, and cubitus valgus in 1938 (75). As a condition that was universally accompanied by short stature, what became known as Turner syndrome was an appropriate candidate for a trial of rhGH therapy. Tanner and his colleagues described the use of pituitary hGH in 6 girls with Turner syndrome in 1971. Treatment was given for only a short time, but seemed promising as growth accelerated while receiving hGH (76). Six patients would be inadequate to advocate the use of hGH in all girls with Turner syndrome. But the stage had been set for others to perform clinical trials to increase height in these girls. In 1979, the Medical Research Counsel Working Party, established in Great Britain to study hGH use in children, did not find any growth response in 9 treated girls with Turner syndrome (77). Rudman and colleagues demonstrated an increase in height velocity when hGH was combined with oxandrolone (78). In 1982, a study including 2 girls with Turner syndrome showed accelerated growth when treated with a combination of hGH and androgen (fluoxymesterone) therapy (79). Despite the conflicting data, by 1983, the Lawson Wilkins Pediatric Endocrine Society (LWPES) and the American Academy Pediatrics were able to state that, “Preliminary data suggest the possibility that such patients [those with Turner syndrome] might benefit from hGH in combination with anabolic steroid therapy or even from rhGH alone,” (80). It was time to turn preliminary data into more formal trials.

The end of the 1980’s brought several clinical trials comparing different hGH regimens. Given previous data that suggested that growth increased when rhGH was administered along with androgens, these larger studies sought to determine if the addition of oxandrolone may increase growth over that induced by rhGH alone. Genentech, as the pioneer of rhGH production, sponsored a study of 70 girls with Turner syndrome randomized to receive their version of rhGH at the time (somatrem, methionyl GH), oxandrolone alone, somatrem plus oxandrolone, or no treatment. The group that received rhGH and sex steroid replacement grew most over the 2 year study period, followed by the oxandrolone group, GH group, and finally, those who had no treatment (81). Later studies tried to determine if adding estradiol to mimic the growth effect of pubertal maturation may have a synergistic effect on the hGH-induced growth response in Turner syndrome. In the short term, addition of estradiol in these studies had a minimal effect on growth when compared to using rhGH alone (82, 83).

But what about adult height? Small short term gains in height velocity were of little use if the women who received treatment would not gain some adult stature. Many of the groups that published data about the short-term effects found with rhGH also followed their patients to near adult height. Height gains between groups were variable as different dosing regimens and sex steroids were used, but adult height increased by 2-8.5 cm over initial predicted adult height when rhGH was administered along with either oxandrolone or ethinyl estradiol (84–88). Treatment also appeared to be safe (89, 90). Finally, the LWPES could state that there were enough data to advocate prescription of rhGH for girls with Turner syndrome (80). FDA approval would soon follow in 1996 (https://www.accessdata.fda.gov/scripts/cder/daf/index.cfm) with follow-up studies corroborating an increase in adult height and normal body proportions when GH was started prior to pubertal induction with or without oxandrolone therapy (91–94).

## Prader-Willi Syndrome (2000)

The first patients of what would later be termed Prader-Willi syndrome (PWS) (OMIM# 176270) were reported in 1956. An article appeared in German describing “A syndrome of obesity, short stature, cryptorchidism, and idiocy in children and adults who presented a myotonia-like picture as newborns” (95). The issue of short stature in this population would not be addressed again for more than 30 years. A preliminary report on the use of pituitary hGH in 4 children with PWS was published in 1987 by Lee and colleagues. They demonstrated an initial increase in height that slowed when hGH was stopped due to the concern of Creutzfeldt-Jakob disease (96). A follow-up report in 2 of the 4 children was published after the children were treated with rhGH and resumed their accelerated growth (97). These studies led to larger trials of rhGH administered for growth promotion in children and adolescents with PWS.

Children with PWS have many features consistent with GH deficiency, including short stature and sub-normal growth, increased truncal fat, and low IGF-1 concentration (98). These findings led to additional studies exploring whether the children actually have GHD (99). Data have demonstrated that children with PWS have hypothalamic dysfunction, including increased risks for ACTH and TSH deficiencies (100). In general, children with PWS have low GH production, as has been determined in subjects with obesity. Levels of IGF-I may be low in children with PWS, however, this contrasts to obese individuals who usually have normal levels and grow normally. Not all children with PWS have altered GH secretion (98) and adult GHD is quite uncommon in the PWS population (101). The reasons for short stature and subsequent positive growth response to rhGH in PWS remain elusive.

Although children with PWS were initially included in studies regarding use of rhGH for short stature, observations began to emerge that suggested that rhGH could increase lean body mass and decrease fat mass (102, 103). This is especially important given the severe obesity that often accompanied a diagnosis of PWS. Larger controlled studies corroborated what was found in the earlier observational studies (104–106). Although published after FDA approval, there have been more recent suggestions that rhGH may improve cognition in children with PWS, if started at a very young age (107–109), although this continues to be debated. In 2000, the FDA had enough information to approve the use of rhGH for children with PWS.

Concerns about rhGH therapy and its relation to adverse outcomes in children with PWS started to emerge within a few years of FDA approval. In Europe, a 6 year old boy with PWS treated with rhGH died suddenly. He had a longstanding history of respiratory pathologies including CPAP-dependence in the neonatal ICU, repeated atelectasis, and pneumonia. However, he developed episodes of sleep apnea only after initiation of rhGH therapy (110). That PWS had been associated with respiratory problems made it difficult to determine a causal relationship between the rhGH treatment and the sudden death in this patient. Additional deaths of patients with PWS in the months following initiation of rhGH continued to emerge (111–113). In 2006, the Pfizer International Growth Database (KIGS) reported 5 children who died suddenly amongst 675 patients with PWS. All died of respiratory disease (114). A trial examining polysomnograms in children with PWS treated with rhGH did not show any increase in obstructive events. However, 1 child, who had had a normal polysomnogram both prior to and after starting GH treatment did die during the trial during a mild respiratory illness (115). The data continue to be conflicting whether sudden death in PWS is inherent to the disease itself or is exacerbated by rhGH treatment. Current rhGH labeling mentions the risk of sudden death in rhGH-treated children with PWS.

## Small for Gestational Age Without Catch-Up to Normal Stature (2001)

Although many children may have short stature, but be born with normal length, certain children start life small and never catch up. The term small- for-gestational age (SGA) has been used to describe those children born smaller than expected. The definition has varied among medical disciplines, but the endocrine community has considered SGA to describe those children born below 2 standard deviations for gestational age and sex for length, weight, or head circumference (116). Children born SGA constitute a heterogeneous group, some have no known underlying medical condition (117). This has made studies of treatment with rhGH in children born SGA with inadequate catch-up growth difficult. Additionally, what constitutes “catch-up” has varied. In the US, the FDA has not defined a minimum height SDS below which rhGH should be considered in SGA children. However, it is generally assumed that patients who do not achieve a length or height that is on the appropriate growth chart for age and sex may be considered for rhGH treatment. In Europe, the criteria are stricter; only children born SGA who remain more than 2.5 SD below the mean for age and sex, who have below average height velocity, and a height SDS more than 1 SD below mid-parental height SDS at age 4 may qualify for rhGH treatment (116). Nevertheless, as a group that had potential to benefit from rhGH therapy, studies were done relatively soon after the use of pituitary hGH showed accelerated linear growth in several SGA subpopulations.

Children with Russell-Silver syndrome are universally born SGA (118–120). In 1969, Tanner and Ham treated 2 children with Russell-Silver syndrome with hGH. Their growth increased, providing a proof of concept that hGH may work, at least in this rare condition (Tanner and Ham, 1969 (121). In the 1970’s, several studies examined hGH treatment in children born SGA who failed to catch up to the normal growth curves including some without a known cause for their small birth size. The response to GH was very variable, with some children increasing in growth similar to those with mild GH deficiency, although others had little to no response (122–124).

It would take until the mid-1990’s for large, multicenter studies to publish data regarding rhGH effects in children born SGA and who failed to catch-up to the normal growth curves. Several studies demonstrated increase in growth and higher predicted or near adult heights in prepubertal children born SGA treated with rhGH (125, 126). Higher doses were needed to achieve increased height velocity as compared to children with GH deficiency (127). In fact, growth occurs in the SGA group in a dose-dependent fashion, with higher doses inducing additional catch-up growth compared to lower doses (128, 129). With the added potential benefit of decreased fat and increased muscle mass (130, 131), the FDA approved the use of GH in children born SGA with inadequate catch-up growth in 2001.

## Idiopathic Short Stature (2003)

Of all the indications for rhGH, ISS is likely the one that has garnered the most debate within the endocrine community. It has brought to the fore such questions as, “Is short stature a disability,” “Is the goal to attain normal height or maximal height,” and, “What exactly are we treating” (132, 133)? Nevertheless, due to the large number of studies that have demonstrated the growth-promoting effect of GH in non-GHD children, the FDA did approve the use of GH in children with ISS is 2003.

In 1984, the first case series attempting to demonstrate a positive effect of hGH in children with ISS was published (134), although there were a number of children who likely had ISS who had been previously treated with hGH in other studies (76). In a study by Grunt and colleagues, seven children with no specific reason for diminished growth were treated with hGH. Five of these patients had an increase in height velocity with a subsequent slowdown after hGH was discontinued (134). This small proof-of-concept study paved the way for pharmaceutical companies, several of which started producing rhGH in the 1980’s after the success of Genentech, to sponsor larger studies to evaluate if children with ISS may benefit from rhGH administration. As expected, the results of the many trials performed from 1989 through the 1990’s demonstrated variable growth results, but the overarching conclusion was that most children with ISS did have an increase in height velocity and (near) adult height when treated with rhGH. This was later corroborated with a meta-analysis performed in 2002. This study evaluated 38 previous reports and concluded that rhGH administration resulted in a 4-6 cm increase over predicted adult height in heterogeneous groups of children with ISS. It was also predicted that the cost per centimeter of treating children with ISS was approximately $US14000, fueling the debates that remain today regarding rhGH administration in this cohort (135).

Why should children with no underlying hormone deficiency respond to rhGH? This, of course, had been asked of other conditions such as Turner Syndrome and children born SGA with inadequate catch-up growth. However, the ISS group has fascinated many since outside of short stature, these children apparently have no other discernable medical issues. In the 1980’s several studies sought to determine the cause or causes of ISS. The first attempts evaluated whether hGH secretion was altered in any way. Several studies, using different methods of GH stimulation and spanning into the 1990’s, were not able show that GH secretion was disrupted in any way in those with ISS (136–138). Later, groups suggested that changes to GH binding protein concentration (139, 140) or function (141, 142) might result in mild GH resistance and accompanying short stature.

The difficulty in studying children with ISS has always been that the cohorts studied are heterogeneous and as time goes on, what constitutes “idiopathic” changes, especially as whole exome and genome sequence determinations become more available. Nevertheless, ISS remains in itself, a common reason for rhGH administration. Because of the controversies regarding treatment of children with ISS with rhGH, the necessity of attempting long-term follow-up for safety monitoring is apparent.

The growth plate itself has become of interest in attempts to disentangle the broad category of ISS. Natriuretic peptide receptor type B, encoded by the *NPR2* gene is intimately involved in the complex regulation of growth. The endogenous ligand is the C-type natriuretic peptide. The prevalence of *NPR2* variants in those with familial short stature (a variety of ISS) was noted as approximately five percent (143).Therapeutic trials with rhGH show increases in C-type natriuretic peptide and its amino terminal pro-peptide (NTproCNP) (143) and accelerated growth (144).

## Short Stature Homeobox-Containing Gene Deficiency Haploinsufficiency (2006)

This indication for rhGH is a perfect example of a condition that was identified and removed from the category “idiopathic” short stature. In fact, in the early 2000’s, several studies demonstrated that SHOX deletions were a relatively common underlying cause for ISS, approximately 2 to 3 percent, although this may be above 10% in selected clinical populations (145, 146). The short stature homeobox-containing gene (SHOX) was first sequenced and suggested as a cause of short stature in girls with Turner syndrome and some children with ISS in 1997 (147). To test the hypothesis of whether children with SHOX haploinsufficiency would respond to rhGH as those with Turner syndrome would, 2 children were treated with rhGH at Turner syndrome doses in 2000. Their growth over 1 year of treatment increased significantly. In fact, over that 1 year, the amount of height gained (+0.9 and +1 SD, respectively) was greater than the mean response in those with Turner syndrome (+0.55SD) (148).

Interestingly, after the association between SHOX haploinsufficiency and Turner syndrome was established, it did not take very long for the FDA to approve rhGH treatment for those children with SHOX deficiency. In fact, the first randomized, controlled trial to be performed using rhGH in children with SHOX haploinsufficiency was not published until 2007, one year *after* FDA approval (149). Since that time, follow-up data have shown that children with SHOX haploinsufficiency started on treatment with rhGH in the prepubertal period can have a height gain of 1.2 SDS (approximately 8 cm) by the time they reach near-adult height (150).

## Noonan Syndrome (2007)

Noonan syndrome is the most recent FDA-approved indication for use of rhGH in children. Jacqueline Noonan first reported on 19 children with similar features including pulmonary stenosis, ptosis, low-set ears, and short stature in 1968 (151). As with Turner syndrome, it was noted over time that children with Noonan syndrome usually had short adult stature and diminished childhood growth (152, 153). As a result, they were prime candidates for an attempt at rhGH treatment. The first report of rhGH treatment in 3 children with Noonan syndrome did not find any increase in height (154). However, several case series in the 1990’s suggested that some patients may benefit from rhGH treatment (155, 156).

In 1996, a large study examining the effect of rhGH in children with Noonan syndrome over 4 years of treatment demonstrated that in most, growth increased, although less than in a group of children with hGH deficiency (157). It wouldn’t be until the next decade that Noonan syndrome was found to be heterogeneous and caused by several genes that resulted in variable phenotypes. Although children with Noonan syndrome had increased growth when receiving rhGH in general, those with the *PTPN11* mutation had the most severe phenotype and responded the least to treatment (158–160).

Before rhGH could be approved for children with the Noonan syndrome, it had to be shown that not only was treatment effective, but it also had to be safe. Given the risk of hypertrophic cardiomyopathy in this population, concerns arose that rhGH may worsen this potentially life-threatening condition. In 1996, a group of 30 patients with Noonan syndrome were treated with rhGH, but did not develop cardiomyopathy. However, this was an observational study without a control group. Additionally, those with pre-existing cardiomyopathy were excluded, not allowing for an examination of whether rhGH worsened this condition (161). In 2001, this missing piece was examined in a study comparing children with Noonan syndrome with underlying heart disease to those who did not. The study group was small and not all had hypertrophic cardiomyopathy, but it was performed over 3 years and did not show any worsening or development of heart disease (162). In 2007, the FDA had enough information to approve the use of GH in children with Noonan syndrome.

A subsequent review of the efficacy and safety of rhGH in Noonan syndrome was performed by Dr. Noonan herself. The review demonstrated an increased height velocity in the first year of treatment and an average near adult height gain of 0.6-1.7 SD when using standardized Noonan syndrome growth curves. Earlier initiation of treatment as well as earlier pubertal status when starting rhGH both seem to be associated with taller near adult height. rhGH appears to be safe in children with Noonan syndrome. The available data reviewed did not demonstrate any exacerbation of cardiac pathology, worsening of glucose metabolism, and no increased risk for cancer. It should be noted, however, that patient numbers in the safety studies reviewed were small necessitating continued monitoring of children with Noonan syndrome who are treated with rhGH (163).

## Some Other Conditions for Which rhGH Has Been Studied, but Do Not Have FDA Approval

Endocrinologists have considered hGH for a variety of children with many conditions for which short stature is a prominent finding. Before the advent of rhGH the supply of hGH was often the limiting factor; however, there were some data to indicate accelerated growth in children with Turner syndrome and small-for-gestational age in a small number of subjects (for example, see Tanner (76, 164). With the supply issue largely moot after the release of rhGH children with multiple other conditions were subjects in trials with rhGH. We have chosen to review data for those with cystic fibrosis, X-linked hypophosphatemia, and achondroplasia (which does have an indication in Japan).

For two other conditions, juvenile idiopathic arthritis (JIA) and inflammatory bowel disease (IBD), mainly Crohn’s disease, significant numbers of children have been treated with rhGH. Both are complex conditions for which marked inflammation (JIA) or inflammation, infection and malabsorption of food (Crohn’s disease) are prevalent. The children with both conditions may be at many stages of their disease process with multiple non-pharmaceutical interventions (e.g., dietary) as well as multiple anti-inflammatory and antibiotic medications, and surgery. Most children receive pharmacological amounts of glucocorticoids. Thus it may be difficult to disentangle the specific effect of rhGH from the myriad other interventions in these complex patients.

Perhaps more narrowly crafted trials might be better suited to test the effects of rhGH (or IGF-1, as some have noted resistance to the effects of rhGH). One might consider, for example, a prospective study with 3 matched study groups of prepubertal children with Crohn’s disease: one with an anti-inflammatory alone (for example, infliximab, anti-TGF-β); anti-inflammatory + rhGH (or rhIGF-1); and the growth factor alone.

Given these constraints and the lack of truly evaluable studies, we have chosen not to summarize them. Although the same may be said about children with cystic fibrosis, the therapy for them is more standardized (non-pharmaceutical as well as antibiotics and pancreatic enzyme replacement). We have chosen to summarize them.

### Cystic Fibrosis (MIM #602421)

Growth in children with cystic fibrosis (CF) has become much more robust and virtually physiologic over the past 5 to 6 decades. Data reported in 1975 noted body weights mostly between -1.0 and -2.0 SD compared to the normal weight curves, but with a sharp descent above age 10 y (165). The data were obtained when the majority of children did not survive into the second decade. Data from Toronto, which had a superb clinic for children with cystic fibrosis, showed that 6% of boys and 12.5% of girls ≥ 8 y were below the 3^rd^ centile for height and 37% of the boys and 40% of the girls were above the 50^th^ centile for height. For weight 8% of the boys and 16% of the girls were below the 3^rd^ centile and 41% of boys and 26% of girls were above the 50^th^ centile. Relevant factors include at first non-pharmaceutical such as physical therapy and nutritional replacement, both macro- and micro-nutrients. Important pharmaceutical agents include anti-bacterial agents, DNAase, pancreatic enzyme formulations, and much more recently the “correctors” such as lumacaftor or tezacaftor and “potentiators” such as ivacaftor (166). These “correctors” and “potentiators” affect the cystic fibrosis transmembrane conductance regulator (CFTR), an ATP-gated anion channel, mutated in those with cystic fibrosis. In the ensuing years the height and weight deficits (compared to physiological growth) have diminished, although most studies report greater catch-up for weight than for height.

Stalvey and co-workers (167) assessed linear growth and weight in 83 children with cystic fibrosis, 6 to 11 y, enrolled in two clinical trials, the longitudinal, observational GOAL study and the placebo-controlled ENVISION study to evaluate the effects of ivacaftor, a CFTR potentiator. Height and weight Z-scores increased over 6 mo (GOAL); height and weight Z-scores increased over 48 weeks (ENVISION) and were greater in the ivacaftor group than in the placebo group—height 7.08 *versus* 5.99 cm; weight 6.45 *versus* 3.34 kg. However the change in the weight Z-score (0.36) was greater than the change in height Z-score (0.17) with the attained weight still at higher Z-score than the height (0.44 *versus* 0.17).

We shall move to clinical trials with rhGH (~0.3/mg/kg/wk) *versus* either an observational group or one with this standard dose *versus* a higher dose (~0.5 mg/kg/wk) group. There were a relatively small number of trials with rhGH. These studies, which are detailed in the Cochrane Library Database of Systemic Reviews (168) are briefly summarized for some pulmonary function, auxologic and blood glucose outcomes, below. Subjects, 291 in total, ages 5 to 23 years were evaluated in 8 clinical trials, all but one at the standard dose of 0.3 mg/kg/wk. The trials were for ~1 year with several for ~6 months. Most of the trial data were limited by low quality of evidence, inconsistency across trials, small numbers of subjects and short duration when considering the entire growth period. The results noted increased height velocity in the intermediate term, but none were taken to (near) adult height.

For those studies for height (n=156) and weight (n=62) and of ~1 y duration the mean HV in the rhGH group was 3.53 cm/y *greater* which attained statistical significance in favor of the interventional group. For weight, the intervention group was 1.0 kg heavier at the end. When the higher dose of rhGH was compared to the placebo the former grew on average 3.3 cm/y more (CI 1.17-5.43 cm). That difference achieved statistical significance. Although the weight gain was 0.80 (CI -0.44 to +2.0 kg) more in the rhGH group, the difference did not achieve statistical significance. These intermediate term data *versus* no treatment showed increases in height and weight, but without a dose response when considering the standard dose *versus* the higher dose. Virtually all showed a small rise in fasting blood glucose levels that were neither statistically nor clinically significant. No subject met the criteria for CFRD or Type II diabetes.

The primary pulmonary outcomes showed no or small changes in FEV_1_ (% predicted) compared to no therapy or with the standard dose compared to the higher dose. Also included are other issues such as pulmonary infections and pulmonary exacerbations and quality of life. All are extensively noted in the Cochrane Database (168).

Taken together these intermediate term data show relatively small changes in height velocity and height SDS as well as for weight. The pulmonary function tests did not increase toward normal but did not show the expected annual decrement of 2 to 3% (169) and there were minor increases in fasting blood glucose level. The level of the evidence was mainly weak using the Cochrane Library Database criteria. Many of the studies were done one and two decades ago when the non-pharmaceutical interventions were being optimized and children with CF were not as well grown as they are today, especially with the newer pharmaceutical interventions. We do not believe that there is a role for rhGH therapy in children and adolescents with CF, whether the end points are pulmonary, infections, metabolic or auxologic.

We believe that a randomized study with matched initial conditions using the best non-pharmaceutical interventions and optimal nutritional and physical therapy interventions as well as non-rhGH pharmaceutical agents such as vitamins, pancreatic enzymes, antibiotics and the “caftors” to test whether standard dose or high dose (perhaps 0.7 mg/kg/week) rhGH might accelerate linear growth and augment lean body mass. Careful consideration of carbohydrate metabolism would be important given the incidence of CFRD.

### X-Linked Hypophosphatemia (MIM #307800)

X-linked hypophosphatemia (MIM #307800) is a rare skeletal dysplasia (prevalence ~1:25,000) featuring renal phosphate wasting and disproportionate short stature. Excessive amounts of fibroblast growth factor 23 (FGF-23) result in hypophosphatemia due to excessive renal phosphate excretion and inappropriately low levels (for the level of phosphate) of 1, 25 di-hydroxy vitamin D (1, 25 [OH]_2_ D]. The result is a skeletal dysplasia (rickets) and growth faltering due in part to a primary defect in osteoblasts (170). Therapy for this condition has changed little over the past few decades, until the approval in 2018 of burosomab, a fully human monoclonal antibody against FGF-23 (171). The antibody is effective by binding to FGF-23 and inhibiting its signaling. It increases renal tubular reabsorption and gastrointestinal absorption of phosphate increasing the serum level of phosphorous and ultimately ameliorates rickets and increases bone mineralization.

To characterize growth faltering in children (n=228) with XLH Mao and colleagues (172) constructed cross-sectional growth curves (for height) of affected children, the vast majority of whom received conventional supplemental therapy with phosphate and an active analog of vitamin D. The subjects were mainly those entered into clinical trials for burosomab and the data indicate their pre-trial stature. In summary, most are born at average length percentiles, but show diminished height velocity within the first year of life. Height velocity and height SDS progressively declined during early childhood and remained deficient thereafter. These data are quite compatible with those of Cagnoli and co-workers (170) who published curves of height for those children evaluated at their clinic, but before any therapy was prescribed.

Segmental growth was affected with leg length decreasing (relatively) progressively during childhood and adolescence. Sitting height, but especially the sitting height index (ratio of sitting height to stature) declined: at age 2 y it was +2 SD, but by age 10 y it had risen to +3.3 SD (173). These findings indicate uncoupled growth of the trunk and legs.

Seikaly and co-workers (174) performed a randomized clinical trial with cross over for rhGH, but in only 5 subjects with XLH. Over 12 months in the treatment group the height Z-score increased to -1.46 from -2.66 SD at the start; there was virtually no change in the control group -2.22 SD compared to -2.27 at the start. The height velocity changed remarkably in the treated group rising to +4.4 SD during rhGH treatment, but remained low (~-1.90 SD) during the control period.

Zivicnjak and colleagues (173) evaluated 16 pre-pubertal children with XLH receiving conventional phosphate and active vitamin D therapy during 3 years in a randomized open-label trial with rhGH administration. For comparison, the same follow-up evaluations were done in a “reference” population of 76 children with XLH receiving the same conventional therapy. At enrollment the children were significantly short (-3.3 SD, for height). Leg length was most impaired (-3.8 SD), and sitting height most preserved (-1.7 SD), yielding a markedly abnormal sitting height index (+3.3 SD). Over the three years of the study there were sustained increases in linear growth (stature, +1.1 SD); sitting height (+1.3 SD) and leg length (+1.3 SD). No significant differences were noted in the controls. These changes including a stable sitting height index remained proportionate in the treated children and controls; however, the sitting height index continued to further increase in the reference population. Eleven patients reached (near) adult height. Their height, sitting height, leg length, and arm length exceeded baseline values by 0.7, 1.7, 0.7, and 1.2 SDS, respectively. Only the sitting height differed significantly from the initial measurements (in SD units).

Other studies on small numbers of subjects have been completed, but not under RCT conditions.

### Achondroplasia (MIM #100800)

Achondroplasia is a common skeletal dysplasia characterized by short stature, rhizomelic shortening of the limbs, trident hands, genu varum, excessive lumbar lordosis and relative macrocephaly. Its genetic basis is a gain of function mutation of the fibroblast growth factor receptor 3 (FGFR3). That permits unbridled proliferation of chondrocytes at the growth plate. The limbs are more affected than the trunk with adult height in the range of -6 to -7 SD compared to the general population. Natural history studies note progressive height deficit as the predominant growth pattern (175).

Once rhGH became available in large quantities children with achondroplasia were considered for therapy. In 1997 Japan became the only country whose medicines approval group (Pharmaceutical and Medical Device Agency) granted a license for use in those with achondroplasia. The first year height velocity was significantly increased over the baseline; however, the increments in the ensuing years were significantly less. Thus, short term growth was more physiologic with a sharp drop-off (**Table 1**). Similar to responses to rhGH in children with other (often mild) skeletal dysplasias, a super-physiological treatment dose led to greater growth.

**Table 1:** Growth in children with achondroplasia treated with rhGH.

	number	Height (SDS)	95% CI
baseline	498	-5.1	-5.1 to -5.0
12 months	494	-4.3	-4.4 to -4.3
24 months	102	-4.1	-4.1 to - 4.0
60 months	21	-3.9	-4.7 to - 3.2

The data are summarized succinctly in a meta-analysis (176). From the selected studies 558 rhGH treated children with achondroplasia were evaluated. The median dosage was 0.21 mg/kg/wk, mainly because most of the studies were done in Japan, where the usual dose of rhGH is closer to 0.16 mg/kg/wk. The baseline height was -5.1 SD that progressively increased during treatment (**Table 1**).

The mean height gain at 60 months, but evaluated only for 21 patients, was 1.1 SD. The height gain stabilized after 24 months.

The effects of rhGH on the disproportion between the limbs and trunk are largely unknown. The same meta-analysis noted above found only 2 studies in which sitting height was properly evaluated (177). It progressively increased over 24 months (n=50 children) from -1.5 SD (95% CI -2.4 to -0.58 SD) to -0.47 SD (95% CI -1.2 to +0.22).

Limb-lengthening surgery may sometimes be performed as well. The children noted in the above analysis (rhGH treatment) did not have such surgery at the time of the study. More recently C-type natriuretic peptide has been evaluated as a potent stimulus of endochondral ossification. Several studies with an analogue of C-type natriuretic peptide have been completed with current long term extensions (178, 179). With this growth promoting agent, children continued to show modest increases in height velocity at least up to 42 months (178) in contrast to the rapid fall in height velocity after the first year in the rhGH clinical trials.

## Long-Acting GH

We have written about the uses of rhGH in children, adolescents, and adults with diagnoses ranging from GH deficiency to various conditions of faltering growth that are responsive to rhGH therapy. A difficult issue relating to rhGH treatment has been the challenge of requiring daily administration of the drug. Adherence to such treatment regimens can lead to large variations in the evaluation of efficacy in any one of the treatment programs that we have discussed above. Thus, the potential availability of long-acting GH (LAGH) preparations, if safe and effective, would be an advantage to overcome the difficulties of requiring daily injections. Two detailed reviews discussing LAGH data (177, 180) and a meta-analysis (181) comparing LAGH to daily treatment provide information about the various formulations that are becoming available. We will briefly describe a few of these.

Speaking generally, there are a number of considerations that require assessment of any one of the products. To achieve satisfactory prolonged duration of action, enabling the delivery of hGH at one, two, or four week intervals, a number of companies have designed new molecular entities that have favorable pharmacokinetic and pharmacodynamic properties. Lack of damage or inflammation at the injection site, slow and steady absorption of the injected material, and reproducible, passage of the modified hGH protein from circulation into GH action sites are requirements. Effective bioavailability with adequate generation of IGF-I is necessary, as well as increased height velocity, which at least mirrors that associated with daily rhGH treatment regimens.

Each preparation differs in its pharmacokinetics, pharmacodynamics, efficacy and safety profile as they are different molecular entities. This makes it difficult to determine when to obtain a level of IGF-1 following the injection to determine the average IGF-1 level as either a safety or efficacy indicator. With daily rhGH one can obtain a level almost any time of any day and be close to a steady state level to make decisions about titration for inadequate efficacy or possible safety issue. For the new, long-acting compounds, one must obtain the full weekly IGF-1 pattern, denote the time when it is close to the average and then one can mathematically determine when the peak is most likely to occur, as well as the average concentration. For example, Kildemoes and colleagues have discussed this consideration for Somapacitan (182).

The authors demonstrated that obtaining a sample for determination of the IGF-I level on day 4 gives an accurate estimate of the mean IGF-I level and determination of IGF-I on day 2 gives an accurate estimate of the of the peak IGF-I level.-The lowest point is usually just before the next weekly injection.

### Depot Preparations

The first LAGH approved in the United States (Genentech; Nutropin Depot) was that of rhGH encapsulated in biodegradable (polylactide-coglycolid) polymer microspheres. There was moderate biological activity with catch-up growth, but some disadvantages of the release of a big burst of rhGH in the first few days following administration as well as sub-therapeutic concentration toward the end of the interval between injections. Troublesome local injection site reactions and issues with large scale production, however, led to discontinuation. In contrast, Declage (Eutropin Plus, Somatropin Biopartners) is produced with rhGH reconstituted in MCT oil and then the resultant hyaluronate microspheres are dissolved by tissue hyaluronidase. Efficacy and safety in non-inferior comparisons to daily rhGH (10.2 cm/y vs 11.1 cm/yr) have been demonstrated leading to approval for use in pediatric GHD for pediatric GHD in South Korea (183).

### PEGylated Preparations

Different polyethylene glycols (PEG), which are biologically inert minimally immunogenic compounds, have been added to GH (PEGylated), among other proteins, to increase their circulatory half-life. Several of the early preparations had local injection site reactions, such as lipoatrophy, along with accumulation of PEG in the choroid plexus cells. These problems led to discontinuation of several products other than Jintrolong, which has been produced by GeneScience Pharmaceuticals (China). Jintrolong is a safe and efficacious drug administered once weekly. It is approved for the treatment of children and adolescents with GH deficiency. The large Phase III trial showed a robust increment in growth leading to approval for use in children with GHD in China (184).

### Prodrug Preparations

TransCon GH (produced by Ascendis Pharma) is a sustained release, unmodified rhGH that is reversibly bound to a PEG carrier molecule *via* a “proprietary” linker. The rhGH is slowly released from this PEG carrier by cleavage of the linker that is temperature and pH dependent with renal excretion. Phase II studies have shown augmented linear growth velocity and increased IGF-I production comparable to that following daily rhGH administration (185). Phase III studies of TransCon GH are underway in young children with GHD and in older patients who had previously been treated with rhGH.

## Non-Covalent Albumin Binding GH

Somacpacitan (produced by Novo Nordisk) reversibly binds to albumin with an increased affinity because of a single amino acid change and thus has a prolonged half-life. Phase II studies showed good toleration of the drug in children who had been small for gestational age infants, along with children with GHD (186). Growth responses in those with GH deficiency are not inferior to those previously treated with daily rhGH. Phase III studies are now being pursued in children and adults with GH deficiency. Use in adults with GH deficiency showed reduced truncal fat and improved body composition which could lead to approval for use in adult GHD and thus, possibly, to off label use in childhood GHD.

### GH Fusion Proteins

A number of proteins have been fused to rhGH to attempt to prolong action by increasing the half-life and delaying renal clearance. HyTropin (Produced by Genexine and Handok of South Korea) is fused to the Fc-domain of immunoglobulin. A Phase II study in Europe demonstrated efficacy and apparent safety in adults with GH deficiency (187).

Somatrogon (produced by OPKO and Pfizer) is a chimeric protein generated by fusing three copies of C-terminal residues of human chorionic gonadotropin beta subunit to the coding sequence of rhGH. A robust growth response found in children treated with Somatrogon was similar to or greater than that seen in prepubertal children with GHD receiving daily rhGH (over 12 cm/yr in a Phase II study) (188). Phase III testing is underway.

## Conclusion

These and presumably other LAGH preparations will become available for use in children, adolescents, and adults in whom hGH treatment is clinically indicated. All of the challenges of determining dosing, modes of administration, methods of monitoring clinical responsivity, and seeking ways of describing long-term safety and efficacy will be as necessary as with daily rhGH treatment. The potential availability of multiple LAGH preparations that could be used will make long-term scrutiny of these GH treatment programs even more difficult than with daily rhGH using basically the same treatments because each is a separate chemical entity and adds different excipients (180).

## Author Contributions

EG drafted and wrote major parts of the manuscript, and reviewed multiple drafts of the entire manuscript. ER drafted and wrote major parts of the manuscript, and reviewed multiple drafts of the entire manuscript. AR conceived the project and drafted and wrote major parts of the manuscript, and reviewed multiple drafts of the entire manuscript. All authors contributed to the article and approved the submitted version.

## Conflict of Interest

The authors declare that the research was conducted in the absence of any commercial or financial relationships that could be construed as a potential conflict of interest.
